# Prevalence and risk factors for latent tuberculosis infection among healthcare workers in Nampula Central Hospital, Mozambique

**DOI:** 10.1186/s12879-017-2516-4

**Published:** 2017-06-08

**Authors:** Celso Belo, Saloshni Naidoo

**Affiliations:** 1 Department of Medicine, Faculty of Health Sciences, Lúrio University, Marrere campus, Street 4250, Km 2.3, Nampula, Mozambique; 2 Discipline of Public Health Medicine, School of Nursing and Public Health, University of KwaZulu-Natal, 2nd Floor, Room 236, George Campbell Building, Howard College, Durban, 4041 South Africa

**Keywords:** Tuberculin skin test, Immunosuppression, Administrative control measures

## Abstract

**Background:**

Healthcare workers in high tuberculosis burdened countries are occupationally exposed to the tuberculosis disease with uncomplicated and complicated tuberculosis on the increase among them. Most of them acquire *Mycobacterium tuberculosis* but do not progress to the active disease – latent tuberculosis infection. The objective of this study was to assess the prevalence and risk factors associated with latent tuberculosis infection among healthcare workers in Nampula Central Hospital, Mozambique.

**Methods:**

This cross-sectional study of healthcare workers was conducted between 2014 and 2015. Participants (*n* = 209) were administered a questionnaire on demographics and occupational tuberculosis exposure and had a tuberculin skin test administered. Multivariate linear and logistic regression tested for associations between independent variables and dependent outcomes (tuberculin skin test induration and latent tuberculosis infection status).

**Results:**

The prevalence of latent tuberculosis infection was 34.4%. Latent tuberculosis infection was highest in those working for more than eight years (39.3%), those who had no BCG vaccination (39.6%) and were immunocompromised (78.1%). Being immunocompromised was significantly associated with latent tuberculosis infection (OR 5.97 [95% CI 1.89; 18.87]). Positive but non-significant associations occurred with working in the medical domain (OR 1.02 [95% CI 0.17; 6.37]), length of employment > eight years (OR 1.97 [95% CI 0.70; 5.53]) and occupational contact with tuberculosis patients (OR 1.24 [95% CI 0.47; 3.27]).

**Conclusions:**

Personal and occupational factors were positively associated with latent tuberculosis infection among healthcare workers in Mozambique.

## Background

The World Health Organization (WHO) reported in 2014 that the African Region had approximately 25% of the global tuberculosis (TB) cases and the highest prevalence of active TB infection (300/ 100,000 cases). Mozambique is classified among the countries with a high burden (countries responsible for 80% of the global burden) of active TB infection (559/ 100,000 cases), fuelled by the number of people infected with human immunodeficiency virus (HIV) [1]. The median prevalence of latent tuberculosis infection (LTBI) among healthcare workers (HCWs) in high income countries is 24% [2] and in low and middle income countries is 54% [3]. There is a correlation between LTBI prevalence among HCWs and regional active TB prevalence. Studies have shown that a high prevalence of active TB infection increases the risk of disease in HCWs [4] with HCWs having a higher probability of getting the disease compared to the general population [2, 5–7].

Most HCWs acquire *Mycobacterium tuberculosis* but they do not present with active disease being in a state referred to as Latent Tuberculosis Infection (LTBI). In this state, the tuberculin skin test (TST) is positive but the clinical and radiological signs are absent. Healthcare workers with LTBI are not infectious but there is a risk of developing active TB if their immunity fails [8].

Studies have shown that there are different risk factors for LTBI in the healthcare centres [5, 9, 10]. Researchers have documented the TB risk factors faced by HCWs worldwide which includes advanced age, sex (male), smoking, years of employment, professional category (physicians and nurses, working closely with patients), delayed diagnosis and misdiagnosis in patients, absence of suspicious clinical signs, lack or inadequacy of personal protective equipment and preventive measures [2, 3, 11–14].

The conversion rate of the TST varies based on the study setting (9.5% to 49.2%) and is higher in middle and low income countries [14–17]. High TST conversion rates mean that there is a significant number of HCWs acquiring LTBI. The median annual incidence of TB infection attributable to healthcare work is 5.8% (range 0–11%) in low and middle income countries and 1.1% (0.2–12%) in high income countries [2]. This highlights the need for surveillance and control measures in healthcare units.

Healthcare units in high TB burden countries still find it difficult to implement TB control measures [18–20]. In Mozambique attempts are being made to screen for LTBI in HCWs in healthcare units considering the high prevalence of HIV in the country and its association with TB. Nampula is the third largest province in Mozambique with a population of 4,529,803 (2011) [21]. Nampula Central Hospital is the only referral tertiary healthcare unit in this area. In the current context of a high population prevalence of TB the aim of this study was to identify the prevalence and risk factors associated with LTBI in HCWs in Nampula Central Hospital.

## Methods

### Study design and population

This cross-sectional study conducted from November 2014 to July 2015 involved HCWs at Nampula Central Hospital, Nampula City, Mozambique. The hospital in the northern region of the country serves a population of approximately 8.5 million in three provinces (*Nampula*, *Cabo Delgado* and *Niassa*) [21]. The hospital has 500 beds with 1200 staff treating approximately 700 out-patients daily [22]. The HCWs in Nampula Central Hospital constituted the study population.

The estimated prevalence of LTBI among HCWs in low and middle income countries is 54% [3]. That is the LTBI prevalence among HCWs used in this study once there was no other suitable value found. Thus, the sample size was *n = 380* using a precision of 5%.

**Fig. 1:**
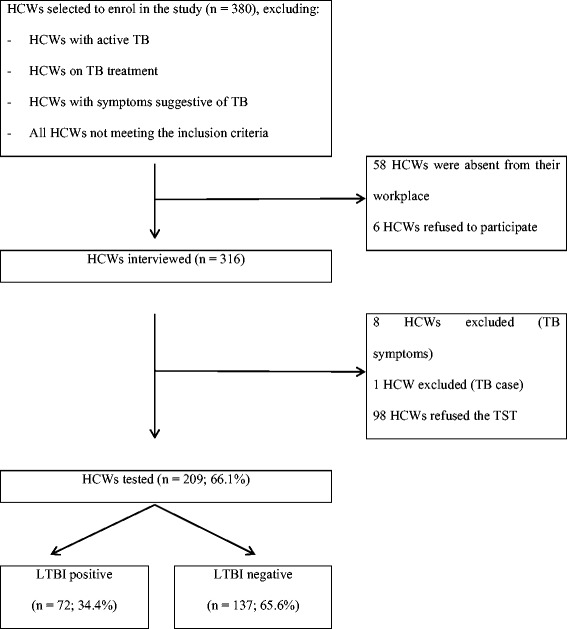
The enrolment process of healthcare workers in the study

All nurses, orderlies and administrators in the hospital were considered for selection in the study. In this way comparisons could be made between groups more and less exposed to TB patients. Doctors were not included in the study due to their reluctance to participate. A complete list of HCWs in each department was obtained from management with the proportion of each category (nurse, orderly and administrator) of HCWs in each department [wards (medicine, paediatrics, surgery, orthopaedics, gynaecology, obstetrics), intensive care unit, outpatient setting, administrative sector] being established. Healthcare workers were randomly selected from the list until the accepted proportion of HCWs per category per department was reached to ensure a good representation of HCWs regarding category and working department. The proportion of HCWs per category and department in the sample was calculated according to the proportion in the list of HCWs collected from the hospital. During the recruitment of the HCWs by the interviewers if a HCW declined participation another one was selected. This process went on until exhaustion of the study population. Unfortunately the sample size was not reached (figure) due to staff refusing to have a second TST. Those with active TB, on treatment and with at least two symptoms suggestive of TB (cough more than two weeks, night sweats, weight loss) were excluded from the study (Fig. 1).

### Data collection (questionnaire)

Initially all volunteering HCWs were administered a TB symptom screening questionnaire [23] with symptomless HCWs being administered a second questionnaire collecting information on individual, occupational and administrative risk factors. This questionnaire of closed ended questions was designed using the TB risk assessment worksheet from United States Centers for Disease Control and Prevention (CDC) [24], validated and corrected in a pilot study. The questionnaire was translated into Portuguese and back translated to ensure important question elements were not lost during translation. Medical students were recruited and trained over five days to perform the interviews. Healthcare workers with TB symptoms were referred to the hospital’s occupational health service for further management.

### Data collection (exposure assessment)

This was based on HCWs response to questions on their exposure to TB and use of administrative control measures. The assessed length of exposure to TB at workplace (patients and co-workers) and at home was at least for 6 months. In this study administrative control measures included cough triage, isolation room, sputum collection and use of personal respiratory protection (PRP) all the time when working.

To diagnose LTBI, HCWs were tested with tuberculin by nurses trained to test TB in patients at Nampula Central Hospital. An intradermal injection of 0,1 mL of tuberculin PPD RT23 was performed using the Mendel – Mantoux technique in the dorsal aspect of the left forearm using a special disposable 1 mL syringe [25]. The test was read forty-eight to seventy-two hours after administration [26]. Those with a positive result were referred to the hospital’s occupational health service for further investigation. Healthcare workers who were negative on first testing were tested two weeks later as per the two-step testing procedure. This procedure is more feasible than a simple TST and commonly used for healthcare personnel screening [24].

The TST results were read as recommended by Jensen et al. where immunity defined TST cut-off points. Immunocompromised HCWs (HIV positive, presence of chronic condition and use of immunosuppressive medication) were positive when TST ≥ 5 mm and non-immunocompromised HCWs when TST ≥ 10 mm [24]. Chronic conditions included immunosuppressive conditions such as diabetes mellitus and cancer. Immunosuppressive medication included chemotherapy and steroids.

Healthcare workers were asked if they knew their HIV status. If answered in the affirmative they were asked if they wish to reveal their status. Healthcare workers who had never been tested for HIV were encouraged to do so. Thirty-one HCWs (14.8%) refused to reveal their HIV status but were retained in the study.

### Data analysis

Data was entered and analysed in SPSS (version 21). The dependent variables were TST induration measured in millimetres and LTBI presence based on the reading of the TST induration and categorization into positive and negative test result (yes/no). The independent variables were age, sex, smoking status, education level, perceived health status, current employment setting, job category, contact with a TB patient at home (last year), duration of employment (years of employment), previous job (last six months), Bacillus Calmette-Guérin (BCG) vaccination, HIV status, chronic conditions and current use of immunosuppressive medication, contact with TB patients, use of PRP and administrative controls practiced at work. Age was categorised in three groups according to the trends found during the data analysis. Healthcare workers reported on their current employment setting in the hospital which was then categorised into work domains (administrative, medical or surgical). The prevalence of LTBI was the proportion of TST positive results of the total number of HCWs tested. Continuous variables were categorised around the mean since data was normally distributed. Means and standard deviations and frequencies were used to describe continuous and categorical variables respectively.

On bivariate analysis the independent-samples *t*-test and ANOVA were used to test for associations between the independent variables and TST induration. Chi square tested for associations between the independent variables and LTBI. Variables were tested for covariance using HCW’s age and working time as covariates (continuous variables) separately, each one at a time, because these two variables are strongly correlated (Pearson value = 0.754).

Multiple linear and logistic regression tested for associations between the independent variables and the continuous and categorical dependent variables respectively while controlling for age and sex. The model was tested controlling for each risk factor to find the one which best explained the influence of risk factors on the dependent variable but the R square value did not improve. The accepted level of significance was 0.05. Based on evidence presented in the literature review all variables from bivariate analysis were entered into the multivariate model. Education level was excluded since it was reflected in the job category. The introduction of variables in the model was done using hierarchical multiple regression starting with age and sex and then each variable was entered in the model by increasing order of the *p*-value.

## Results

### Participant demographics

Three hundred and sixteen (83.2%) of 380 HCWs, participated in the questionnaire survey. Two hundred and nine HCWs consented to have a TST, ninety eight refused and nine were excluded based on exclusion criteria. The mean age of HCWs was 36.8 years (standard deviation (SD) 7.8) with a female predominance (68.4%). There was no significant difference between the HCWs consenting to the TST (*n* = 209) and those who refused (*n* = 98) with respect to demographic and occupational variables shown in Table 1.

**Table 1:** Demographics of healthcare workers from Nampula Central Hospital (*n* = 316)

	Participants interviewed & tested			Participants who refused TST	
	N (SD)	% [range]	Prev of LTBI	N (SD)	% [range]
General					
Sex					
Total	209	100	34.4	96^a^	100
Male	66	31.6	34.8	40	41.7
Female	143	68.4	34.3	56	58.3
Mean age, years	36.8 (7.8)	[23–56]		36.5 (8.7)	[23–59]
Age					
≤ 32	69	33.2	29.0	34	34.7
33–40	75	36.1	38.7	32	32.7
> 40	64	30.7	35.9	32	32.7
Current smoking					
No	201	96.2	34.8	95	96.9
Yes	8	3.8	25.0	3	3.1
Race					
Black	201	98.6	34.8	96	100.0
Coloured	3	1.4	0.0	0	0.0
Marital status					
Married	104	50.0	36.5	52	54.7
Single	95	45.7	31.6	40	42.1
Divorced	3	1.4	33.3	0	0.0
Widow	6	2.9	33.3	3	3.2
Level of education					
Primary	36	17.2	38.9	24	24.5
Secondary	152	72.7	33.6	66	67.3
Post-secondary	21	10.0	33.3	8	8.2
BCG vaccination					
No	48	27.3	39.6	19	22.1
Yes	128	72.7	31.3	67	77.9
Perceived health status					
Poor	111	53.4	36.0	49	50.0
Good	97	46.6	32.0	49	50.0
Immunosuppression					
No	177	84.7	26.6	79	80.6
Yes	32	15.3	78.1	19	19.4
Contact with TB patient at home					
No	164	78.5	33.5	74	75.5
Yes	45	21.5	37.8	24	24.5
Housing type					
Urban house	178	85.6	34.8	83	84.7
Hostel	0	0.0	0.0	0	0.0
Squatter	1	0.5	100.0	0	0.0
Rural house	29	13.9	31.0	15	15.3
People living at same house					
≤ 6	115	55.0	32.2	61	62.9
> 6	94	45.0	37.2	36	37.1
Occupational					
Occupational category					
Administrative staff	50	23.9	36.0	7	7.1
Orderly	91	43.5	33.0	56	57.1
Nurse	68	32.5	35.3	35	35.7
Employment setting					
Administrative	38	18.2	28.9	15	15.3
Medical domain	96	45.9	38.5	43	43.9
Surgical domain	75	35.9	32.0	40	40.8
Mean of working time, years	10.2 (8.3)	[0.8–33]		10 (8.7)	[1–35]
Working time					
≤ 8	119	57.2	31.1	57	58.8
> 8	89	42.8	39.3	40	41.2
Contact with TB patients at workplace					
No	87	41.6	29.9	34	35.1
Yes	122	58.4	37.7	63	64.9
Administrative control measures					
No	141	71.2	30.5	66	70.2
Yes	57	28.8	38.6	28	29.8
Co-worker TB positive					
No	154	82.8	33.8	76	88.4
Yes	32	17.2	34.4	10	11.6
Past history of TB					
No	2	40.0	0.0	2	100.0
Yes	3	60.0	0.0	0	0.0

^a^Missing two participant data on age

**Table 2:** Crude association of demographics, occupational factors and tuberculin skin test measurement (*n* = 209)

	N	TST measurement (millimetres)		*p*-value
		Mean	SD^a^	
Sex				
Male	66	7.18	3.258	0.892
Female	143	7.26	4.036	
Age				
≤ 32	69	7.20	3.567	0.392
33–40	75	7.65	4.206	0.759
> 40	64	6.77	3.809	0.786
Current smoking				
No	201	7.24	3.735	0.934
Yes	8	7.13	5.515	
Level of education				
Primary	36	7.64	4.001	0.313
Secondary	152	7.30	3.697	0.877
Postsecondary	21	6.10	4.158	0.302
Employment setting				
Administrative	38	6.87	3.699	0.795
Medical domain	96	7.27	3.948	0.846
Surgical domain	75	7.37	3.694	0.784
Occupational category				
Administrative staff	50	7.02	3.485	0.868
Orderly	91	7.37	4.122	0.858
Nurse	68	7.21	3.614	0.963
Working time, years				
≤ 8	119	7.30	3.903	0.802
> 8	89	7.17	3.693	
BCG vaccination				
No	48	7.00	3.531	0.857
Yes	128	7.12	3.960	
Perceived health status				
Poor	111	6.88	3.531	0.176
Good	97	7.60	4.071	
Contact with TB patients at workplace				
No	87	6.85	3.572	0.218
Yes	122	7.51	3.946	
Administrative control measures				
No	141	6.75	3.717	0.029
Yes	57	8.04	3.722	
Immunosuppression				
No	177	7.35	3.792	0.301
Yes	32	6.59	3.843	
Contact with TB patient at home				
No	164	7.30	3.882	0.641
Yes	45	7.00	3.516	
People living at same house				
≤ 6	115	7.06	4.111	0.466
> 6	94	7.45	3.391	
Co-worker TB positive				
Yes	154	7.28	3.897	0.969
No	32	7.25	3.331	

^a^Standard deviation

**Table 3:** Multiple linear regression of the demographics, occupational factors and tuberculin skin test

	Coefficient β	95% CI^a^	*p*-value
Age (years)	−0.05	−0.16; 0.11	0.712
Sex			
Male	-	-	-
Female	0.00	−1.48; 1.56	0.962
Administrative control measures	0.16	−0.15; 2.86	0.077
Perceived health status	0.07	−0.80; 1.92	0.415
Contact with TB patients at workplace	0.03	−1.22; 1.74	0.727
Immunosuppression	−0.06	−2.44; 1.27	0.533
People living at same house (> 6)	0.07	−0.83; 1.88	0.443
Contact with TB at home	−0.03	−1.80; 1.31	0.761
Working time (years)	−0.02	−0.01; 0.01	0.874
Employment setting			
Administrative	-	-	-
Medical domain	0.08	−1.95; 3.15	0.644
Surgical domain	0.06	2.22; 3.19	0.722
BCG vaccination	−0.02	−1.62; 1.34	0.851
Current smoking (yes)	0.02	−3.07; 3.66	0.863
Occupational category			
Administrative	-	-	-
Orderly	−0.02	−2.50; 2.20	0.900
Nurse	−0. 06	−3.02; 2.01	0.694
Co-worker TB positive	−0.03	−2.08; 1.40	0.701

^a^Confidence Interval (CI)

**Table 4:** Associations between demographics, occupational factors and latent tuberculosis infection using multiple logistic regression

	Crude Odds Ratio 95% CI^a^	Adjusted Odds Ratio^a^ 95% CI^b^	*p*-value
Age			
≤ 32	Reference	Reference	0.380
33–40	1.55 [0.77; 3.10]	0.83 [0.30; 2.29]	0.712
> 40	1.37 [0.66; 2.85]	0.42 [0.11; 1.56]	0.194
Sex (female)	1.01 [0.83; 1.23]	0.75 [0.30; 1.92]	0.552
Administrative control measures	0.78 [0.50; 1.21]	1.33 [0.53; 3.31]	0.539
Perceived health status (Good)	1.10 [0.80; 1.51]	0.99 [0.43; 2.27]	0.988
Contact with TB patients at workplace	0.87 [0.69; 1.09]	1.19 [0.45; 3.14]	0.725
Immunosuppression	0.15 [0.07; 0.32]	5.82 [1.84; 18.39]	0.003
People living at same house (> 6)	0.89 [0.65; 1.20]	0.92 [0.40; 2.14]	0.853
Contact with TB at home	0.87 [0.51; 1.47]	1.22 [0.48; 3.12]	0.680
Working time (>8 years)	0.82 [0.60; 1.12]	1.91 [0.68; 5.38]	0.224
Employment setting			
Administrative	Reference	Reference	0.983
Medical domain	1.54 [0.68; 3.47]	1.02 [0.17; 6.31]	0.980
Surgical domain	1.23 [0.49; 2.71]	0.95 [0.15; 5.85]	0.952
BCG vaccination	1.11 [0.90; 1.36]	0.66 [0.26; 1.65]	0.373
Current smoking (yes)	1.58 [0.33; 7.61]	0.00	0.999
Occupational category			
Administrative	Reference	Reference	0.771
Orderly	0.87 [0.42; 1.80]	0.55 [0.11; 2.81]	0.476
Nurse	0.97 [0.45; 2.08]	0.56 [0.10; 3.17]	0.511
Co-worker TB positive	0.98 [0.50; 1.90]	0.93 [0.32; 2.67]	0.887

^a^Adjusted for age and sex; ^b^Confidence Interval (CI)

### Prevalence of LTBI

The LTBI prevalence among tested HCWs was 34.4% (*n* = 72, [0.28;0.41] 95% Confidence Interval (CI)). Healthcare workers aged 33 to 40 years had the highest prevalence of LTBI (*n* = 75; 38.7%). Non-smokers HCWs had a higher LTBI prevalence (*n* = 201; 34.8%). Lower educated HCWs had a higher LTBI prevalence (*n* = 36; 38.9%). The LTBI prevalence was higher in HCWs with no previous BCG vaccination (*n* = 48; 39.6%) and immunocompromised HCWs (*n* = 32; 78.1%).

Healthcare workers in the medical domain had the highest prevalence of LTBI (*n* = 96; 38.5%) compared with the surgical domain and administrators. The prevalence of LTBI was higher among HCWs who worked for more than eight years (*n* = 89; 39.3%), in the presence of administrative control measures (*n* = 57; 38.6%) and in those who reported contact with TB patients at the workplace (*n* = 122; 37.7%) (Table 1).

### Associations of independent variables with TST induration

On bivariate analysis TST induration was significantly greater among HCWs who were exposed to administrative controls (mean: 8.04 cm) compared to those who were not (mean: 6.75 cm) (*p* = 0.029) (Table 2). On multivariate linear regression no significant associations were found (Table 3).

Adjusted for age and sex

### Associations of independent variables with LTBI presence (Table 4)

Healthcare workers aged 33 years to 40 years (Odds Ratio (OR) 1.55 [95% CI 0.77; 3.10]) and more than 40 years (OR 1.37 [95% CI 0.66; 2.85]) had higher odds of having LTBI as compared to the younger group on bivariate analysis even though not significant. There was a positive association between being smoker and LTBI (OR 1.58 [95% CI 0.33; 7.61]) with being female positively associated with LTBI (OR 1.01 [95% CI 0.83; 1.23]). Administrative control measures had a negative association being protective (OR 0.78 [95% CI 0.50; 1.21]) in bivariate but positive (OR 1.33 [95% CI 0.53; 3.31]) in multivariate analysis.

On multivariate analysis there was no significant association found with LTBI with the following risk factors but positive association were demonstrated: working in the medical domain (OR 1.02 [95% CI 0.17; 6.31]), working for more than eight years (OR 1.91 [95% CI 0.68; 5.38]), contact with TB patients in the workplace (OR 1.19 [95% CI 0.45; 3.14]) and at home (OR 1.22 [95% CI 0.48; 3.12]). On multivariate analysis, being immunocompromised (OR 5.82 [95% CI 1.84; 18.39]) was significantly associated with a diagnosis of LTBI. BCG vaccination showed a negative association with LTBI which was not significant (OR 0.66 [95% CI 0.26; 1.65]).

## Discussion

This study of LTBI prevalence among HCWs in Mozambique provides valuable information in a country classified as a high TB burden country by the WHO in 2013 [1]. Studies have shown that there are different risk factors that contribute to the transmission of TB in health care centres [5, 9, 10]. Healthcare workers are a group with a higher probability of acquiring TB rather than the general population [5], hence the importance of this study’s findings. The prevalence found in this study (34.4%) was similar to the LTBI prevalence found among HCWs in South Korea [27]. The only study conducted in Mozambique on LTBI among HCWs reported a prevalence of 41% in high-risk group (HIV-positive with TST ≥ 5 mm) and 18% in low-risk group (HIV-positive with TST < 5 mm OR HIV-negative with TST between 10 and 14 mm) [28]. Although ninety eight (31.0%) HCWs refused the TST there were no significant difference in occupational and demographic characteristics between the two groups limiting the impact of selection bias and potential confounding on LTBI prevalence (Table 1).

The prevalence of LTBI was higher in HCWs more than 32 years of age (and highest in age group 33–40 years) with a positive association on bivariate analysis although not statistically significant. There are other studies which have reported a high prevalence with advancing age [27, 29, 30, 31]. A possible reason for the high LTBI prevalence seen among the 33 to 40 year old in this study may be that this age group has worked in the hospital environment for a sufficiently long time to develop an immune response to TB while the younger HCWs are just entering the environment and may not have been sufficiently exposed to mount an immune response.

The prevalence of LTBI was very similar between males and females (34.8% vs 34.3% respectively).

Contrary to what was expected from the literature [32, 33] a high prevalence of LTBI was found amongst non-smoking HCWs (34.8%). This can be explained by the very small portion of smokers in the sample (*n* = 8, 3.8%) and presence of immunocompromised amongst the non-smokers.

Immunosuppression is a very important individual risk factor with a high LTBI prevalence (78.1%) and a statistically significant association (OR 5.82 [95% CI 1.84; 18.39]). The wide confidence interval may be reflective of our small sample size however Van Rie et al. found HIV associated with a high prevalence of LTBI and an increased probability of progression to TB disease [13]. This has important implications for HCWs who may be living with HIV and are at increased risk of developing TB. This also requires important management decisions with respect to allocation of HCWs who are living with HIV to work domains in the hospital in order to ensure minimal occupational risk for developing active TB. The treatment of healthcare workers with LTBI using isoniazid is other aspect to be considered.

In this study the prevalence of LTBI was higher in the HCWs who had not been vaccinated with BCG. This is supported on multivariate analysis where a protective relationship was shown with BCG vaccinated HCWs being less likely to have LTBI (OR 0.66 [95% CI 0.26; 1.65]. The relationship between BCG vaccination and LTBI varies among studies [33, 34]. In our study, the probability of BCG vaccine confounds TST result is very remote once the vaccine is given at birth in Mozambique.

Healthcare workers working in the medical domain reported a higher prevalence of LTBI compared to the surgical domain similarly to what Tan et al. found [35]. Other studies found a similar pattern among the occupational categories [36]. The lack of significant association between being a nurse and having LTBI on multivariate analysis is similar to reports in other studies [37, 38].

Being employed for more than eight years was positively associated with having LTBI (OR 1.91 [95% CI 0.68; 5.38]. Costa et al. found similar results in their study using a TST cut-off point ≥10 mm [31]. This would suggest that the risk of LTBI increases with employment exposure.

The high prevalence of LTBI (37.7%) and positive association on multivariate analysis (OR 1.19 [95% CI 0.45; 3.14]) seen in HCWs in contact with TB patients at work is consistent with work by Whitaker et al. that concluded that HCWs more often in contact with TB patients have a higher LTBI prevalence [30]. In Mozambique there is a high burden of undiagnosed TB patients in healthcare facilities which undoubtedly increases the occupational risk for LTBI among HCWs. The use of administrative control measures had a protective trend with LTBI (OR 0.78 [95% CI 0.50; 1.21]). Besides LTBI status (negative/ positive) as dependant variable TST induration measurements was used as continuous variable making possible to analyse trends in the means [39].

The major limitation of our study is related with TST disadvantages (less specific for diagnosis of LTBI than blood tests using IGRA and dependent on the technician who performs the test) and definition of immunosuppression since the conditions were self-reported (chronic condition, HIV-positive and immunosuppressive medication) and not clinically validated. The TST limitations have contributed to the high refusal rate especially the number of laboratory visits to perform and read the test as the two-step testing method was used. Other limitations include: no doctors among the study participants, self-reported TB exposure (questionnaires), self-reported BCG vaccination, self-reported HIV status and lack of LTBI prevalence in the general population for comparison purpose. Doctors were reluctant to participate in the study mainly related to the number of laboratory visits to comply with two-step TST.

## Conclusions

In conclusion, amongst the risk factors for LTBI in our study we found positive, though non-significant, associations with increasing age, being female, working in the medical domain, working for a longer duration in healthcare and contact with TB patients at work and home. Immunosuppression was significant on multiple logistic regression analysis. Immunosuppression is largely related to HIV [40]. The prevalence of LTBI in HCWs with a potential risk for active TB disease exists in developing countries as seen in this study in Mozambique. Implementation of infection control practices and medical surveillance for HCWs in Mozambique is required to monitor and prevent LTBI conversion to active TB disease.

## Abbreviations

BCG: Bacillus Calmette-Guérin
CI: Confidence interval
HCW: Healthcare worker
HCWs: Healthcare workers
HIV: Human Immunodeficiency Virus
LTBI: Latent Tuberculosis Infection
OR: Odds ratio
PRP: Personal Respiratory Protection
SD: Standard deviation
TB: Tuberculosis
TST: Tuberculin Skin Test
WHO: World Health Organization
